# Protective distal side-to-side neurorrhaphy in proximal nerve injury—an experimental study with rats

**DOI:** 10.1007/s00701-019-03835-2

**Published:** 2019-02-12

**Authors:** Henrikki Rönkkö, Harry Göransson, Hanna-Stiina Taskinen, Pasi Paavilainen, Tero Vahlberg, Matias Röyttä

**Affiliations:** 1 Department of Orthopaedics, Kanta-Häme Central Hospital, Hämeenlinna, Finland; 2 Department of Hand and Microsurgery, Tampere University Hospital, Tampere, Finland; 3 Department of Hand Surgery, Turku University Hospital, Turku, Finland; 4 Institute of Clinical Medicine, Department of Biostatistics, University of Turku, Turku, Finland; 5 Department of Pathology, University of Turku, Turku, Finland

**Keywords:** Peripheral nerves, Nerve injury, Chronic denervation, Nerve repair, Nerve regeneration, Side-to-side repair

## Abstract

**Background:**

Side-to-side neurorrhaphy may protect the denervated end organ and preserve the initial connection with proximal stump. We examined the effect of protective side-to-side anastomosis on nerve and end organ regeneration in proximal nerve injury model.

**Methods:**

The left common peroneal nerve of 24 Sprague Dawley rats was proximally transected. In groups B and C, side-to-side neurorrhaphy was performed distally between the peroneal and tibial nerves without (group B) and with (group C) partial donor nerve axotomy inside the epineural window. Group A served as an unprotected control. After 26 weeks, the proximal transection was repaired with end-to-end neurorrhaphy on all animals. Regeneration was followed during 12 weeks with the walk track analysis. Morphometric studies and wet muscle mass calculations were conducted at the end of the follow-up period.

**Results:**

The results of the walk track analysis were significantly better in groups B and C compared to group A. Groups B and C showed significantly higher wet mass ratios of the tibialis anterior and extensor digitorum longus muscle compared to group A. Group C showed significantly higher morphometric values compared to group A. Group B reached higher values of the fibre count, fibre density, and percentage of the fibre area compared to group A.

**Conclusions:**

Protective distal side-to-side neurorrhaphy reduced muscle atrophy and had an improving effect on the morphometric studies and walk track analysis. Distal side-to-side neurorrhaphy does not prevent the regenerating axons to grow from the proximal stump to achieve distal nerve stump.

## Introduction

The regenerative results have often been unsatisfactory in proximal nerve injuries. This has been considered a consequence of the degenerative changes of the nerve due to the delay of the growing axons to reach their end organs. The number of Schwann cells begins to decline, and their capacity to secrete neurotrophins decreases [15, 21, 27, 29, 38]. Basal lamina tubes begin to degrade and will be substituted with connective tissue [25, 35]. At the same time, muscle atrophy proceeds because of lack of reinnervation.

It has been presumed that timely reinnervation of the distal nerve and end organ could solve the problem. Distal end-to-end (ETE) [2, 9, 22, 33, 34, 37] and end-to-side (ETS) [3, 4, 7, 8, 12, 16–18, 31, 32] nerve transfers and free nerve grafts [14, 20, 36] have been used.

Distal side-to-side (STS) anastomosis has been shown to have regenerative potential clinically in the treatment of proximal nerve injuries [6, 39, 40] However, to our knowledge, there are no experimental comprehensive studies dealing with side-to-side anastomosis in proximal nerve injuries in international literature. This method leaves the distal nerve free for further reconstructions. It is potentially advantageous, especially in cases where regeneration from the proximal stump of the injured nerve is expected. In addition, the repair can be performed in one operation and there is no need for nerve grafts. This systematic experimental study was conducted to examine the ability of the distal side-to-side nerve anastomosis to give time to proximal regenerating nerve to grow distally avoiding degeneration of the distal nerve and muscle. In our model, delayed end-to-end repair was used to simulate the proximal injury.

We hypothesise that protective distal side-to-side anastomosis has an improving effect on the results of walk track analysis and nerve morphometry and reduces muscle atrophy related to proximal nerve injury. We also presume that intentional donor nerve axotomy inside side-to-side anastomosis improves the repair results.

## Materials and methods

### Animals

Twenty-four female young adult Sprague Dawley rats (Central Animal Laboratory, University of Turku, Finland) weighing 223–300 g were randomly divided into three experimental groups. The animals were allowed to receive laboratory chow and drink tap water ad libitum. The temperature was kept at 22 °C ± 2 °C, and the humidity was 50% ± 10%. The day cycle in the animal room was constant (lights on from 6:00 AM to 6:00 PM). There were no wound infections during the experiment. The National Animal Experiment Board approved all interventions, the analgesic treatment, and animal care (ESLH200901886/Ym-23, the decision STH168A).

### Operative procedure

**Fig. 1:**
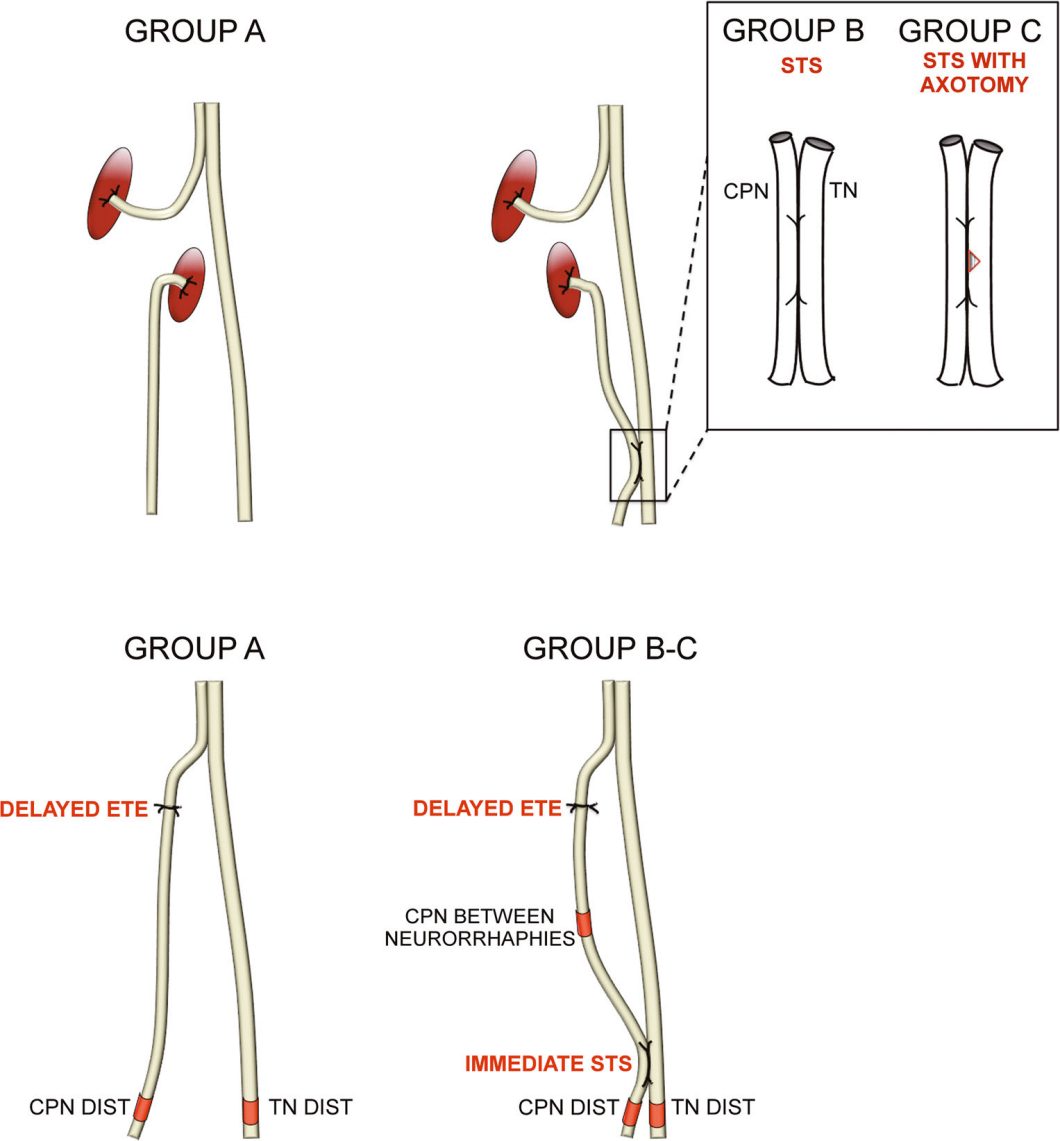
Schematic representation of interventions. In the first operation (upper panel), in all groups, the common peroneal nerve (CPN) is proximally transected and left unrepaired. In group B, distal side-to-side (STS) neurorrhaphy is performed without intentional axonal injury to the nerves. In group C, donor axonal injury to the extent of half of the nerve is done to the donor nerve inside the epineural windows and side-to-side anastomosis is performed similarly to the previous group. After 26 weeks, the second operation (lower panel) is performed similarly in all groups; proximal injury is repaired in an end-to-end fashion. The red marks show the nerve biopsy sites

In the first operation under medetomidine hydrochloride 5 μg/kg (Domitor, Orion Oyj, Espoo, Finland) and ketamine hydrochloride 750 μg/kg (Ketalar, Pfizer Oy, Helsinki, Finland) anaesthesia, the left common peroneal nerve (CPN) was ligated with two sequential 8–0 sutures (Nylon, S&T AG, Neuhausen, Switzerland) 5 mm distally from the site of bifurcation of the left common peroneal nerve and tibial nerve (TN) on all animals (Fig. 1). Transection of the common peroneal nerve was done between the ligations, and the nerve ends were turned in opposite directions and sutured in the adjacent muscle with three 10-0 sutures (Nylon, S&T AG, Neuhausen, Switzerland) to prevent reinnervation. In group B, an additional distal side-to-side anastomosis was performed with 2-mm-long epineural windows in both common peroneal nerve and tibial nerve and sutured with four 11-0 microsurgical nylon knots (Monosof, Covidien, Mansfield, MA, USA) under surgical microscopy Wild M3Z (Wild Leitz Ltd., Heerbrugg, Switzerland). In group C, the operation was similar to group B, but in distal side-to-side anastomosis, an axotomy to the extent of half of the donor tibial nerve was performed. Group A served as an unprotected control. In all animals, the muscle layer and skin were closed in separate layers with 5–0 absorbable sutures (Bondek Plus, Deknatel, TFX Medical Ltd., High Wycombe, UK). Perioperatively, the animals were injected subcutaneously 5 ml sodium chloride 9 mg/ml (Fresenius Kabi AB, Uppsala, Sverige) to maintain fluid balance. A subcutaneous injection 5 mg/kg carprofen (Rimadyl, Vericode Ltd., Dundee, UK) was given to animals during three postoperative days as an analgesic drug.

After 26 weeks, a second operation was performed on all animals. Normal appearance, construction, and consistence of nerve ends were ensured by recurrent sharp excisions of neuroma formations. Proximal injury of common peroneal nerve was repaired with tension-free end-to-end anastomosis with four 11-0 microsurgical nylon knots (Monosof, Covidien, Mansfield, MA, USA) under surgical microscopy. The same investigator carried out all operations. The follow-up time was 12 weeks after the second operation.

### Walk track analysis

The walk track analysis was performed preoperatively, and 2, 4, 6, 8, 12, 16, 20, and 26 weeks after the first operation, and 2, 4, 6, 8, and 12 weeks after second operation. The animals walked freely at least six times per time point in a steady velocity along an 80 × 115 × 515-mm corridor with a darkened box at the end. Only walks without stops were included in analysis. Ink staining from both hind limbs was analysed. The print length (PL, distance between heel and third toe), toe spread (TS, distance between first and fifth toe), and intermediate toe spread (IT, distance between second and fourth toe) were calculated as a mean of three measurements. The peroneal function index (PFI) was calculated with following formula: PFI = 174.9((EPL − NPL)/NPL) + 80.3((ETS − NTS)/NTS) − 13.4 [1]. “N” refers to the normal, unoperated side, and “E” refers to the experimental side The investigator has passed the self-education test of the walk track analysis [5] and was blinded to know the intervention groups during analysis.

### Sample preparation

Terminal anaesthesia was induced with an intraperitoneal injection of sodium pentobarbital 60 mg/kg (Mebunat, Orion Oyj, Espoo, Finland). After perfusion of formalin, operated nerves were harvested. The sites of nerve samples of common peroneal nerve and tibial nerve taken to the morphometric analysis are shown in Fig. 1. Common peroneal nerve–innervated tibial anterior and extensor digitorum longus and tibial nerve–innervated gastrocnemius muscles were carefully dissected from both legs with the microsurgical technique and weighed with a balance PG403-S DeltaRange (Mettler–Toledo GmbH, Greifensee, Switzerland). Tissue samples were immersion-fixed in phosphate-buffered formalin for overnight. Four-micrometre-thick sections were cut from paraffin blocks for haematoxylin and eosin staining or immunocytochemistry.

### Neurofilament protein immunocytochemistry

The stainings were performed with a biotin-free Poly-HRP-Anti-Mouse kit (BrightVision, Immunologic BV, Duiven, The Netherlands) according to the manufacturer’s instructions. Mouse monoclonal neurofilament (200 kDa and 68 kDa) Ab1 (Clone 2F11) antibody (Thermo Fisher Scientific, Fremont, CA, USA) was applied and incubated. Normal Antibody Diluent (Immunologic BV, Duiven, The Netherlands) was used to dilute and stabilise HRP-conjugates. The sections were incubated with peroxidase-compatible chromogen (Bright-DAB, Immunologic BV, Duiven, The Netherlands) for 8 min and finally counterstained and coverslipped.

### Morphometry

Neurofilament-stained whole-nerve cross-sections were photographed with an AxioCam HRc camera (Carl Zeiss, Göttingen, Germany) connected to an AxioVert 200 M microscope (Carl Zeiss, Göttingen, Germany). The mosaic images were adjusted with AxioVision software (Carl Zeiss, Jena, Germany). Imaging software (Graphics Suite X6/Photo-Paint, Corel Comp., Ottawa, ON, Canada) was used to process subepineural areas of digitalized images. With BioImageXD software [19], the following morphometric outcomes were analysed: nerve area (μm^2^), fibre count, fibre area (μm^2^), total fibre area (μm^2^), fibre density (n/mm^2^), and percentage of fibre area (total fibre area/nerve area × 100; Table 1).

**Table 1:** Results of morphometric analyses of common peroneal nerve and tibial nerve

	Nerve area (μm^2^)	Fibre count	Mean fibre area (μm^2^)	Total fibre area (μm^2^)	Fibre density (n/mm^2^)	Percentage of fibre area (%)
Common peroneal nerve						
Between neurorrhaphies						
Group B	58,047 (19618)	1613 (468)	3.7 (0.50)	6100 (2208)	29,044 (6616)	10.7 (2.3)
Group C	77,098 (26166)	2810 (711)	4.0 (0.35)	11,236 (3078)	37,395 (4354)	14.9 (2.8)
Distal						
Group A	29,497 (15773)	353 (239)	3.0 (0.66)	1088 (856)	13,242 (9176)	4.0 (2.9)
Group B	57,434 (14409)	1573 (305)	4.5 (0.80)	7042 (1875)	27,869 (3714)	12.3 (1.2)
Group C	88,926 (38971)	3044 (706)	5.4 (0.59)	16,277 (5175)	36,819 (7280)	19.1 (3.4)
Intact control	148,517 (26881)	2313 (102)	20.3 (2.7)	46,998 (7461)	15,943 (2310)	31.9 (3.4)
Tibial nerve						
Distal						
Group A	436,961 (104329)	5527 (278)	20.0 (4.6)	110,704 (27063)	13,165 (2575)	25.4 (3.2)
Group B	295,626 (49259)	5234 (488)	13.9 (2.0)	71,962 (6849)	17,951 (2149)	24.8 (4.0)
Group C	244,208 (57916)	4973 (651)	10.9 (2.2)	53,692 (9747)	20,963 (3665)	22.3 (2.8)
Intact control	372,928 (84512)	5212 (500)	20.8 (2.7)	108,870 (21318)	14,409 (2323)	29.4 (2.2)

Data is expressed in terms of mean (SD)

### Statistical analysis

The statistical analysis was done with the help of an experienced statistician with SPSS software (version 24, IBM Corp., Armonk, NY, USA). In our earlier study, the side-to-side repair group with donor axotomy reached a peroneal function index value of 35.7 (9.08) at 12 weeks postoperatively. The sample size of eight animals per group gives 90% power and a type I error rate of no more than 5% to detect a difference of 15 or more in the mean peroneal function index values between groups. Differences with *p* < 0.05 were considered statistically significant. The data are expressed in terms of mean (SD).

Type 3 tests of fixed effects were used to reveal significant differences between the intervention groups. The comparisons between the groups in the results of the walk track analysis were analysed with the covariance analysis ANCOVA for repeated measurements which was adjusted to the baseline peroneal function index values. Inside every group, the value before second operation and the value at the end of the follow-up period were compared to reveal the effect of second operation. The Tukey–Kramer adjustment was used to control the effect of multiple comparisons.

In the morphometric analysis, the groups were compared with the one-way ANOVA variance analysis with Tukey–Kramer adjustment for multiple comparisons. If the global *p* value was significant, we have made pairwise comparisons between groups using Tukey’s method. Comparison of two different biopsy sites of the same nerve was performed with the paired *t* test. The fibre area values were normally distributed after log10-transformation. The linear mixed model with Tukey–Kramer and Dunnett adjustments was used to compare the nerve fibre area values.

The wet mass ratios were compared using the Mann–Whitney *U* test with Bonferroni adjustment for multiple comparisons. The correlations between the peroneal function index, wet mass ratios, and morphometric outcomes were calculated with Pearson correlation coefficients.

## Results

### Walk track analysis

**Fig. 2:**
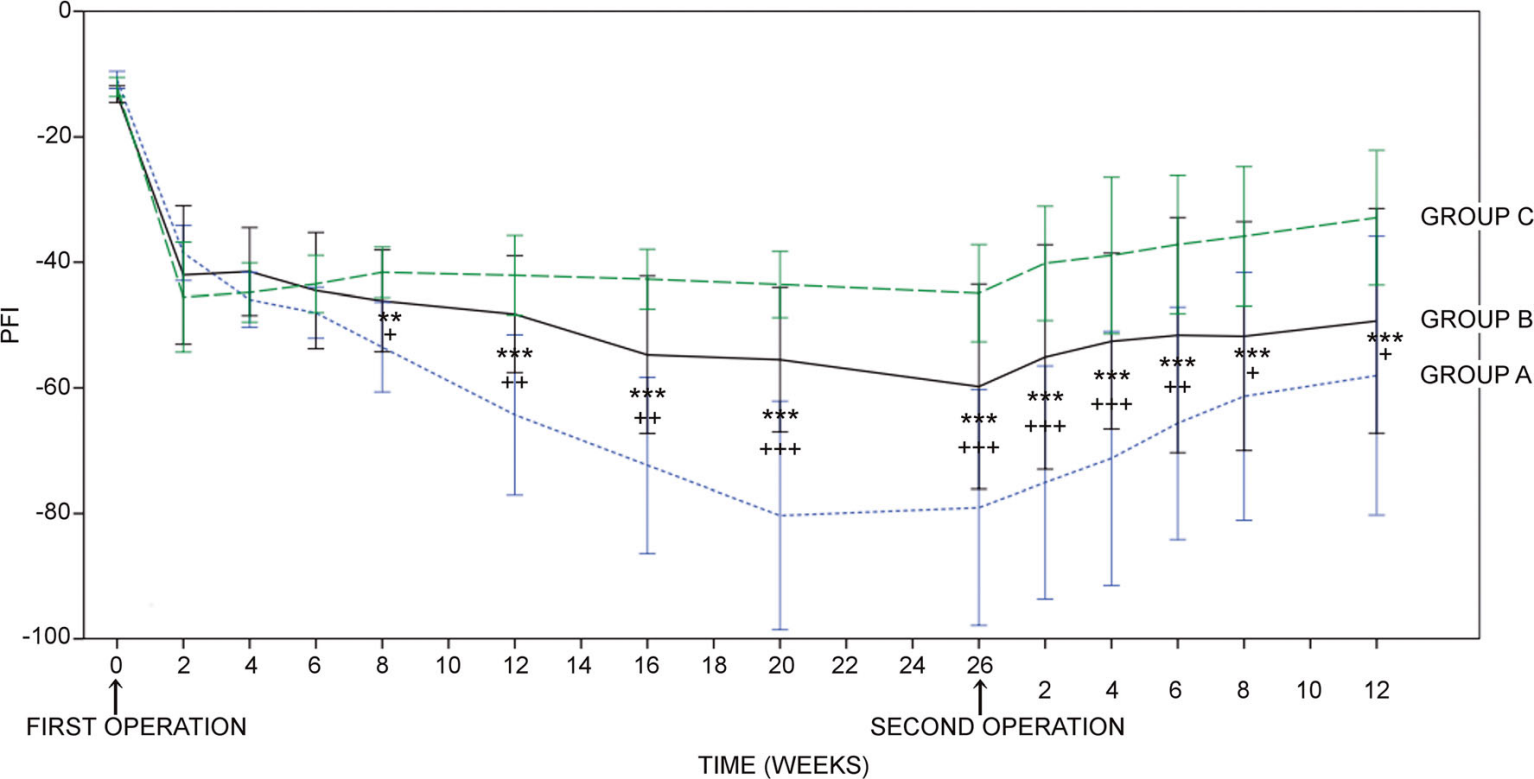
Results of the walk track analysis. Groups B and C show significantly higher peroneal function index (PFI) values compared to group A from 8 weeks onward. There are no significant differences between groups B and C. The data are analysed with the analysis of covariance with Tukey–Kramer adjustment for multiple comparisons. *p < 0.05, **p < 0.01, ***p < 0.001 comparison of group A to C, ^+^*p* < 0.05, ^++^*p* < 0.01, ^+++^*p* < 0.001 comparison of group A to B. Error bar, ± 1 SD

Groups B and C reached significantly higher peroneal function index values compared to group A from 8 weeks onwards to the end of the follow-up time (Fig. 2). Groups B and C did not differ. When comparing the peroneal function index values at 26 weeks before the second operation to the peroneal function index values at the end of the follow-up, there were significant increases in all groups (group A, *p* < 0.001; B, *p* = 0.007; and C, *p* = 0.008).

### Morphometry

#### Common peroneal nerve (Table 1)

##### Distal sections of common peroneal nerve (Fig. 1, lower panel)

**Fig. 3:**
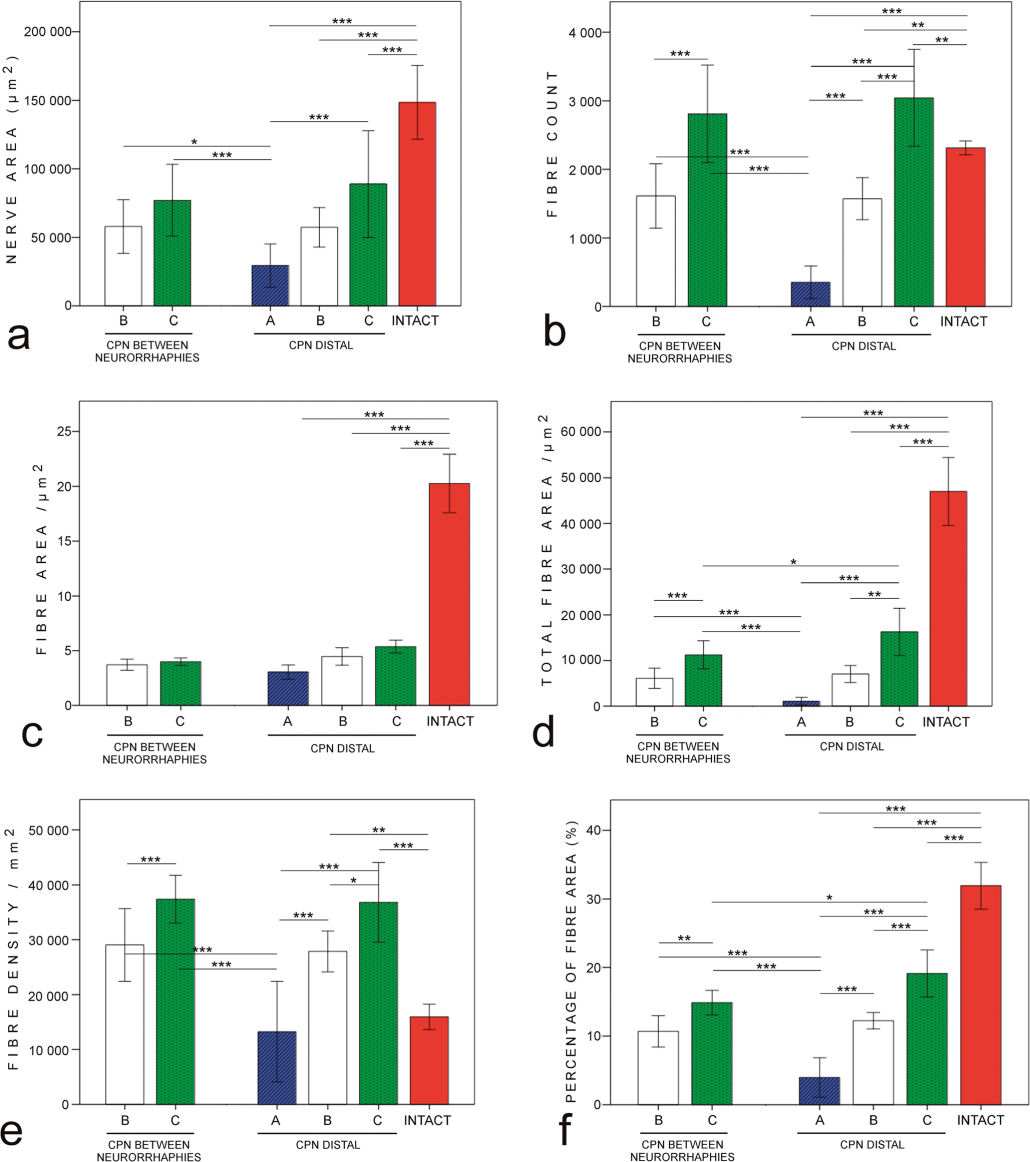
Morphometric results of the common peroneal nerve. Group C shows statistically significantly higher values of all morphometric outcomes except mean fibre area compared to group A. Group B reaches higher values of fibre count (**b**), fibre density (**e)**, and percentage of the fibre area (**f**) compared to group A. When comparing groups B and C, group C shows higher values of fibre count (**b**), total fibre area (**d**), fibre density **(e**), and percentage of the fibre area (**f**) both at the site between neurorrhaphies and distal to common peroneal nerve. *p < 0.05, **p < 0.01, ***p < 0.001. Bars express the mean values, error bar ± 1 SD

All morphometric outcomes except mean fibre area of group C showed significantly higher values compared to group A (all *p* < 0.001) (Fig. 3a–f). In group B, the fibre count, fibre density, and percentage of the fibre area reached significantly higher values compared to group A. The fibre count, total fibre area, fibre density, and percentage of the fibre area values were higher in group C compared to group B (all *p* ≤ 0.03). The intervention groups did not reach the nerve area, total fibre area, and percentage of the fibre area values of the intact group (all *p* < 0.001). The fibre count and fibre density values of group C showed higher values compared to the intact group (both *p* ≤ 0.004). The mean fibre area values of the intact group were higher compared to three intervention groups (all *p* < 0.001). There were no significant differences between the groups A, B, and C.

##### The specimens between neurorrhaphies (Fig. 1, lower panel)

Group C showed higher values of fibre count, total fibre area, fibre density, and percentage of the fibre area compared to group B. Groups B and C reached higher values of all morphometric outcomes compared to the distal specimens of group A (all *p* ≤ 0.03).

When comparing two different biopsy sites, the total fibre area, and the percentage of the fibre area values of group C were higher in the distal sections compared to the sections between the neurorrhaphies (both *p* ≤ 0.04). In group B, the morphometric values of two biopsy sites did not differ significantly.

#### Tibial nerve (Table 1)

Distal specimens of tibial nerve were studied (Fig. 1, lower panel). The fibre count and the percentage of the fibre area values of the intervention groups did not differ significantly. The mean fibre area and total fibre area values of group A were higher compared to group C (both *p* < 0.001) but not to group B. Group A did not differ compared to the intact group in any morphometric outcome. The mean fibre area, total fibre area, and percentage of the fibre area values of the intact group were significantly higher compared to groups B and C (all *p* < 0.04).

### Light microscopy

#### Distal common peroneal nerve

**Fig. 4:**
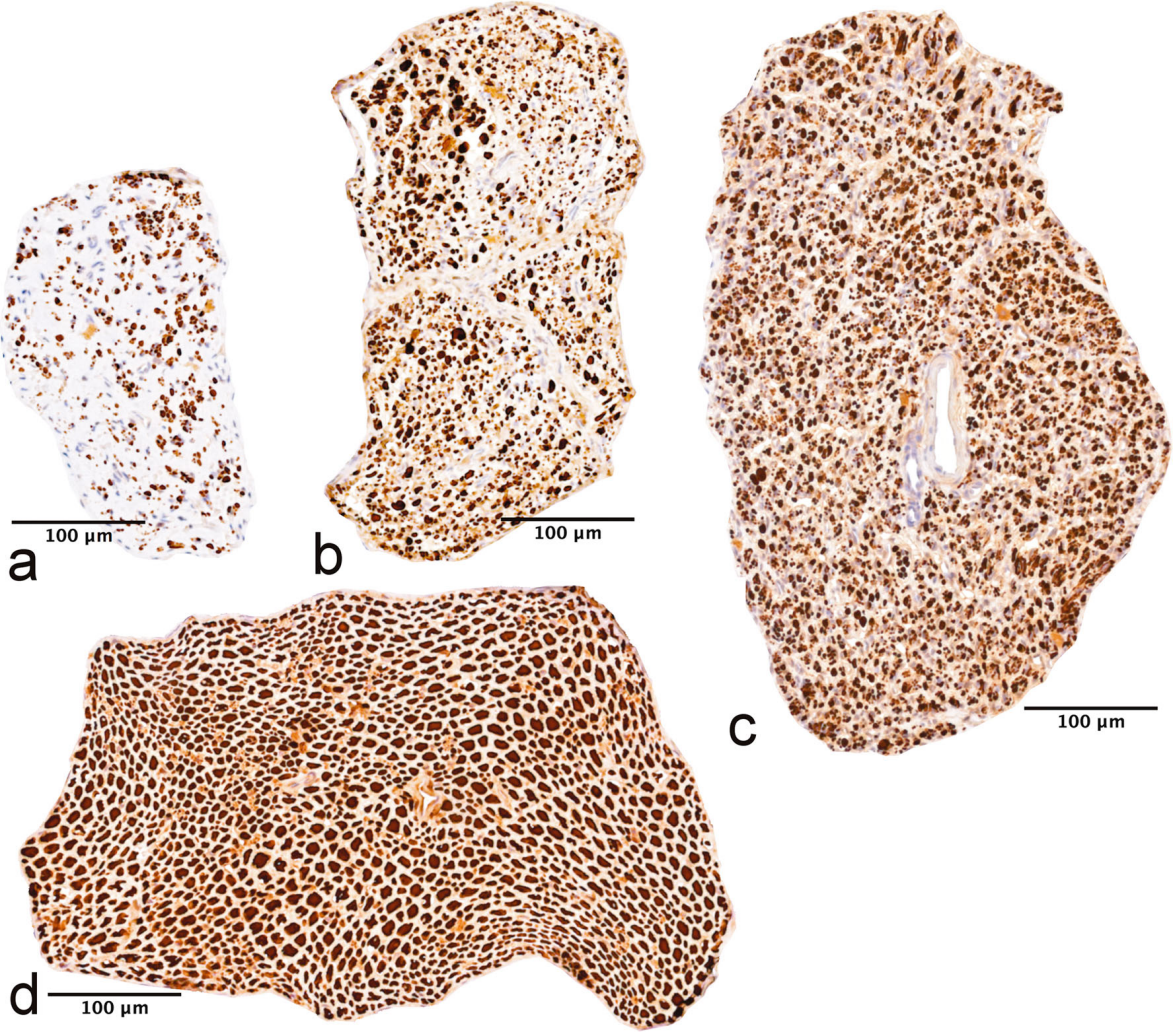
Nerve sections of the distal common peroneal nerve. Morphometric analyses are done with whole-nerve cross-sections. The nerve structure is better preserved in groups B (**b**) and C (**c**) compared to group A (**a**). In groups B and C, the fibre density is high, but the fibre area is smaller compared to intact nerve (**d**). Neurofilament staining

In group A, both the nerve area and the individual axons looked smaller compared to the other groups, and in some sections, there were connective tissue in the subepineural area (Fig. 4).

#### Common peroneal nerve between neurorrhaphies

The axonal regeneration was more robust in group C compared to group B. In both groups, some axon sprouts were seen outside the epineurium.

#### Distal tibial nerve

In group A, the sections looked normal. Occasionally, in groups B and C, some axons were seen outside the epineurium. In some sections of group C, the axons were smaller in the lateral zones.

#### Tibialis anterior and extensor digitorum longus muscles

In group A, there were clear signs of general atrophy with small, angular-shaped cells. In groups B and C, the size and shape of muscle cells were generally normal.

#### Gastrocnemius muscle

In all groups, the general appearance was normal, but in groups B and C, mild focal signs of atrophy were detected.

### Muscle mass calculations

**Fig. 5:**
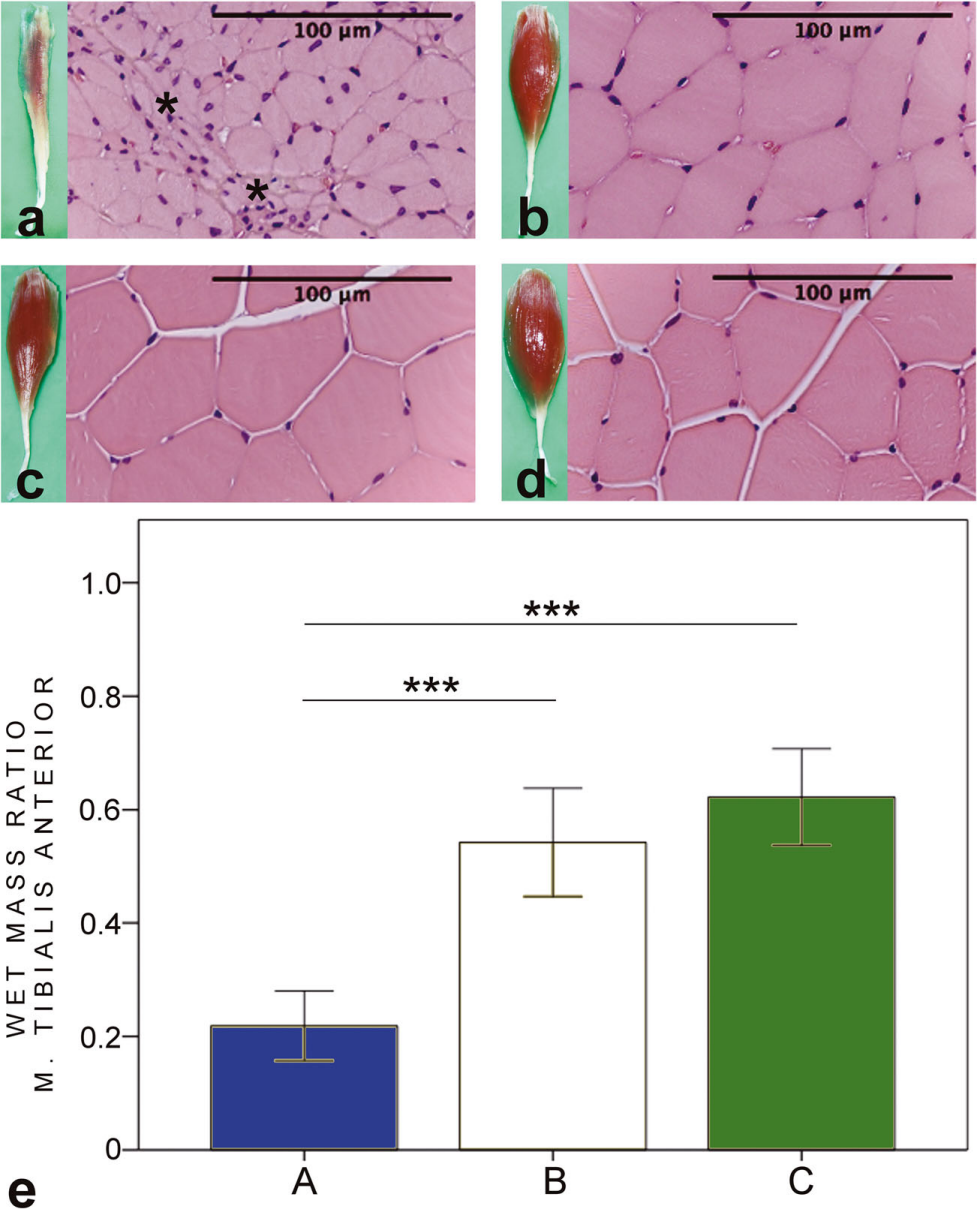
HE-stained biopsies and macroscopic figures of the tibialis anterior muscle of the intervention groups (**a–c**) and control sample from the contralateral side (**d**). In group A (**a**), the muscle fibres are small and angular-shaped (asterisk). In groups B and C, focal signs of atrophy can be seen. The wet mass ratio (**e**) is significantly higher in groups B and C compared to group A. In group A, the wet muscle mass value of tibialis anterior (TA) muscle is 22 (6.2)% of the contralateral side value. In groups B and C, the values are 54 (9.6)% and 62 (8.5)%, respectively. *p < 0.05, **p < 0.01, ***p < 0.001. Bars express the mean values, error bar ± 1 SD

**Fig. 6:**
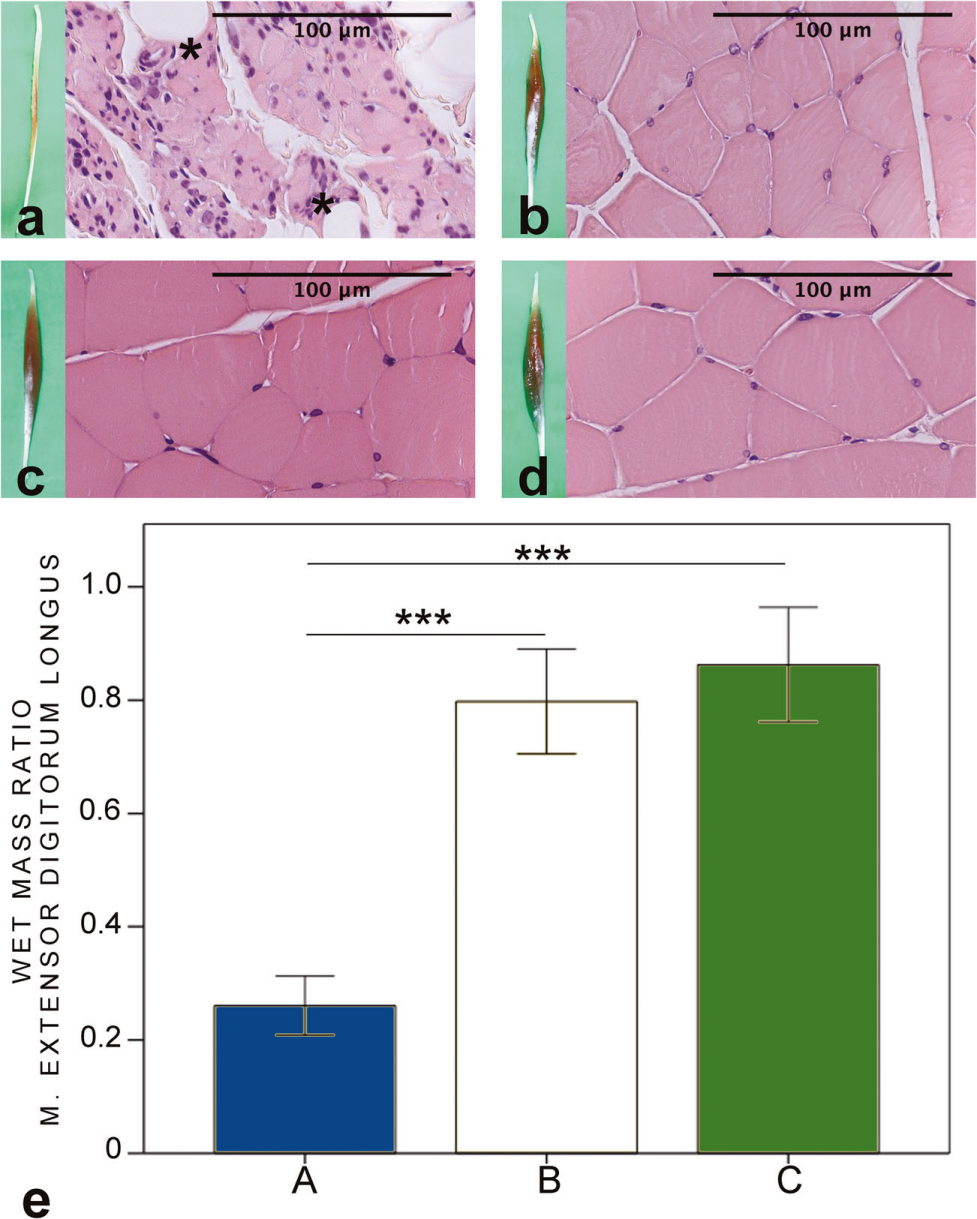
HE-stained biopsies and macroscopic figures of the extensor digitorum longus muscle of the intervention groups (**a–c**) and control sample from the contralateral side (**d**). Clear signs of atrophy and replacement of adipose tissue (asterisk) are seen in group A. In groups B and C, the muscle cells are large, but there are focal signs of atrophy. The wet mass ratio (**e**) is significantly higher in groups B and C compared to group A. In group A, the wet muscle mass value of extensor digitorum longus (EDL) muscle is 26 (5.2)%, in group B 80 (9.3)%, and in group C 86 (10.1)% of the contralateral side values. *p < 0.05, **p < 0.01, ***p < 0.001. Bars express the mean values, error bar ± 1 SD

**Fig. 7:**
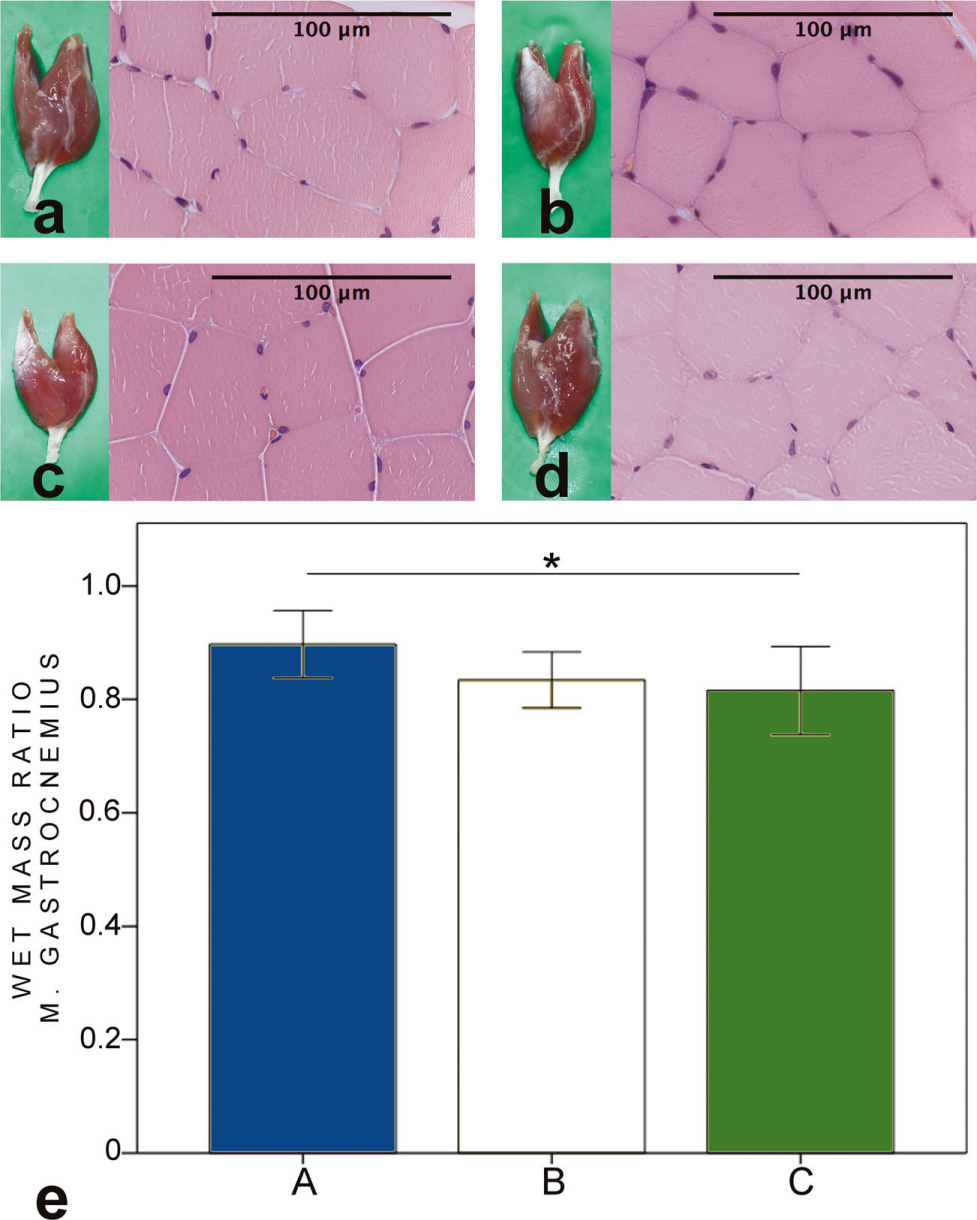
HE-stained biopsies and macroscopic figures of the gastrocnemius muscle of the intervention groups (**a–c**) and control sample from the contralateral side (**d**). Muscle architecture was well preserved in all groups. However, mild focal signs of atrophy were detected in groups B and C. In wet mass ratio calculations (**e**), group A (89 (6.0) %) got higher values compared to group C (82 (7.8) %). Groups B (83 (4.9) %) and C did not differ. *p < 0.05, **p < 0.01, ***p < 0.001. Bars express the mean values, error bar ± 1 SD

In groups B and C, the wet mass ratio of the tibialis anterior muscle (Fig. 5) and extensor digitorum longus muscle (Fig. 6) were significantly higher compared to group A (both *p* < 0.001). The wet mass ratio of the gastrocnemius muscle (Fig. 7) was significantly higher in group A compared to group C (*p* < 0.05), whereas there were no significant differences between groups A and B and groups B and C.

### Correlations between outcomes

The morphometric parameters of distal common peroneal nerve, the wet muscle ratios of the tibialis anterior and extensor digitorum longus muscles, and the corresponding peroneal function index values of the walk track analysis at the end of the follow-up period correlated significantly with each other. Morphometric parameters of distal common peroneal nerve correlated with peroneal function index value at 12 weeks: nerve area (Pearson correlation 0.41, *p* = 0.05), fibre count (0.63, *p* < 0.001), fibre area (0.54, *p* = 0.006), total fibre area (0.57, *p* = 0.003), fibre density (0.74, *p* < 0.001), and percentage of fibre area (0.71, *p* < 0.001). Significant correlations were found also with wet muscle ratios of tibialis anterior and extensor digitorum longus muscles: nerve area (both Pearson correlations ≥ 0.55, both *p* ≤ 0.005), fibre count (≥ 0.78, *p* < 0.001), fibre area (≥ 0.78, *p* < 0.001), total fibre area (≥ 0.73, *p* < 0.001), fibre density (≥ 0.80, *p* < 0.001), and percentage of fibre area (≥ 0.89, *p* < 0.001). Peroneal function index value at 12 weeks correlated with wet mass ratios of tibialis anterior and extensor digitorum longus muscles (both ≥ 0.65, *p* < 0.001).

## Discussion

The regeneration capacity of the proximal peripheral nerve injury is a multilevel problem. The success of proximal nerve repair depends both on the capacity of regenerating axons to arrive to distal stump [23] and the capacity of the distal nerve stump to support the neural regeneration before muscle atrophy occurs. Fu and Gordon [10, 11] showed that prolonged denervation of Schwann cells in the distal stump is more detrimental to nerve regeneration than prolonged axotomy of the proximal stump. Furthermore, in case of proximal injury, the repair can always be considered delayed due to the lengthened time it takes to reach the end organs.

In the present study, immediate distal side-to-side neurorrhaphy near the end organs was performed to reduce muscle atrophy and to maintain the growth-supportive environment in the distal nerve stump. Protective distal side-to-side anastomosis (groups B and C) had an improving effect on the results of the morphometric studies, muscle wet mass, and walk track analysis (PFI) compared to unprotected delayed end-to-end nerve repair (group A).

In morphometry, the parameters of both side-to-side groups differed significantly compared to unprotected end-to-end repair. In our previous study, side-to-side repair without deliberate axotomy produced mean 881 fibres to the distal common peroneal nerve at 26 weeks representing the innervation of immediate side-to-side anastomosis to the recipient nerve [24]. In the present study, there were mean 1573 fibres in the distal common peroneal nerve in group B representing the combined innervation of both the immediate side-to-side anastomosis and delayed proximal end-to-end repair. In group A, the fibre count was mean 353 after mere delayed proximal end-to-end repair. Thus, it can be assumed that the immediate side-to-side anastomosis did not prevent the ingrowth of the regenerating axons from the proximal end to reach the distal nerve stump after delayed end-to-end repair. The mechanism controlling axonal regeneration in distal nerve stump is not totally understood. We know that without regeneration, the denervated Schwann cells change their phenotype from growth-supportive to myelinating progressively after 1 month [15, 21, 38]. Exogenous cytokines have been used to mimic interactions between macrophages and Schwann cells and to restore the growth-promoting capacity of Schwann cells [13, 28]. In the side-to-side bridge model, Schwann cells in the denervated recipient nerve are shown to be able to change their phenotype after reconstruction to a proliferative phenotype and again to redifferentiate to a myelinating phenotype when the donor axon has grown into the recipient nerve [14].

In groups B and C, the wet mass ratios of the common peroneal nerve–innervated tibialis anterior, and the extensor digitorum longus muscles, were significantly higher compared to group A. Distal side-to-side anastomosis could clearly reduce muscle atrophy. Our result is in relation to the study of Ladak et al. [11]. They reported with 4-month delayed repair a 1.6-fold increase in the tibialis anterior wet muscle weight at a 5-month follow-up compared to the unprotected group [20]. In our study, the increase was 2.5-fold. Although the fibre count values were significantly higher in group C when compared to group B, the wet mass ratios between the groups did not differ. Additional donor nerve axotomy inside side-to-side anastomosis (group C) did not significantly increase the recipient wet mass ratios compared to side-to-side neurorrhaphy without donor axonal injury (group B). A different number of axons preserved muscle mass at the similar level. It is known that denervated muscle fibres can partly compensate the reduced number of motor units by increasing the size of motor units [11, 30]. Denervated muscle fibres are able to receive reinnervation to some extent, but they are not able to recover to the earlier diameter [11]. This was seen in the present study with delayed end-to-end repair (group A), as the peroneal function index values improved with limited axonal flow, but the muscle atrophy remained considerable. The tibialis anterior muscle wet mass ratio (0.22) of group A was equal compared to unrepaired group after 32 weeks denervation (0.21, our unpublished data).

The results of the walk track analysis showed that in groups B and C, the recovery was twofold. The first improvement was seen after the distal side-to-side repair and the second after the delayed proximal end-to-end repair. This suggests that both the immediate side-to-side repair and the delayed proximal end-to-end repair have influence on the functional outcome, and thus, both repair procedures are worth performing. The results are in accordance with the previous so-called baby-sitting techniques [3, 4, 7, 8, 12, 16–18, 31, 32].

When evaluating the effects of partial donor nerve axotomy on recipient nerve, also, the effects on donor nerve have to be assessed. In the present study, side-to-side with mere epineural window did not have significant deleterious effect on donor nerve. However, the impairing effect was seen with side-to-side protection with deliberate partial donor nerve axotomy. The mean fibre area and total fibre area values were higher in the unprotected group (group A) compared to the side-to-side protection with donor nerve axotomy (group C). Also, the muscle mass ratio of gastrocnemius muscle was higher in unprotected group (group A, 89.7%) compared to side-to-side protection with donor nerve axotomy (group C, 81.5%) (Fig. 7). The side-to-side protection with epineural window and unprotected group did not have significant differences in the results of donor nerve morphometry or wet muscle mass ratios.

Distal side-to-side anastomosis may provide a useful tool to reconstruct proximal complete or incomplete nerve injuries. It does not sacrifice the distal end of the injured nerve or a healthy adjacent nerve. However, a prerequisite for this technique is the appropriate anatomical structure that allows the approximation of two parallel nerve trunks. For cases not allowing this, various other techniques have been developed to connect parallel nerves to the side-to-side bridge technique with nerve grafts [14, 20, 36] or synthetic conduits [26]. The obvious weaknesses of the present study are the differences in the regeneration capacity as well as the differences of the nerves between rodents and humans. In the morphometric analysis, the regeneration of motor axons could not be separated from sensory axons, and the neurofilament staining does not allow the measurement of myelin sheath. In further studies, retrograde labelling techniques and electron microscopy are warranted.

## Conclusion

The effect of early distal side-to-side anastomosis was studied in the proximal nerve injury model. Distal side-to-side neurorrhaphies were able to reduce muscle atrophy. Side-to-side anastomosis with partial axotomy improved the morphometric results, but did not have a significant effect on the recipient nerve innervating muscle mass amounts or the results of the walk track analysis compared to bare side-to-side anastomosis. Protective distal side-to-side neurorrhaphies did not impair axon growth from the proximal stump to the distal nerve after delayed proximal end-to-end neurorrhaphy. Both immediate side-to-side anastomosis and delayed end-to-end repairs contributed to the improved results of the walk track analysis. This technique may have potential to improve the results in high-level nerve injuries.
